# Controlled Evaluation of Hybrid Multi-Face Recognition Pipelines for Real-Time Occluded Face Recognition on Edge Devices

**DOI:** 10.3390/s26134069

**Published:** 2026-06-26

**Authors:** Shkëmb Abdullahu, Arbana Kadriu, Marco Piangerelli

**Affiliations:** 1 Department of Computer Science, Faculty of Contemporary Sciences and Technologies, South East European University, 1200 Tetovo, North Macedonia; a.kadriu@seeu.edu.mk; 2 Computer Science Division, School of Science and Technology, University of Camerino, 62032 Camerino, Italy; 3 R&D Division, Vici & C. S.p.A., 47822 Santarcangelo di Romagna, Italy

**Keywords:** partially occluded face recognition, multi-face recognition, SCRFD, ArcFace, ResNet100, linear SVM, Real-World Occluded Faces dataset, real-time recognition

## Abstract

Accurate recognition of partially occluded faces remains challenging in unconstrained and real-time environments, especially under masks, partial occlusions, pose variation, and illumination changes. This study presents a controlled comparison of three hybrid multi-face recognition pipelines for robust occluded face recognition. For fair evaluation, all pipelines use the same SCRFD face detector, preprocessing protocol, Linear SVM classifier, and 60% unknown rejection threshold, while varying only the feature extractor: ResNet29, ConvNeXt, and ResNet100 with ArcFace embeddings. To reduce data leakage, models are trained only on normal, non-occluded faces and tested on unseen partially occluded faces. Evaluation is performed on a custom dataset and the public Real-World Occluded Faces dataset, alongside three existing paper methods with publicly available code tested under the same experimental protocol. The SCRFD with ArcFace ResNet100 and Linear SVM pipeline achieved the best results compared to existing papers and our other pipelines, reaching 97.475% real-time accuracy for five faces and over 99% confusion-matrix-based accuracy on the custom dataset. On the ROF dataset, it also achieved closed-set accuracies of 98.66% for sunglasses and 97.92% for masks, with threshold-based accuracies of 96.35% for the sunglass test and 95.14% for the mask test. Furthermore, it obtained EER values below 0.007 and AUC values above 99%. In real-time testing, it achieved 29.25 FPS with 34.18 ms/frame latency on a GPU-enabled laptop and approximately 5 FPS with 273.4 ms/frame latency on a Raspberry Pi 4.

## 1. Introduction

Face recognition has become an important component of camera-based sensing systems used in access control, surveillance, attendance monitoring, identity verification, and edge-oriented Internet of Things (IoT) applications [1]. In such systems, visual data are acquired through cameras or embedded imaging sensors and processed through a sequence of stages, including face detection, preprocessing or alignment, feature extraction, and identity classification. Although deep learning-based face recognition models have achieved high accuracy under controlled conditions, their performance can still decrease significantly in unconstrained environments where faces are affected by partial occlusion, pose variation, illumination changes, and background complexity.

Partially occluded face recognition remains a challenging problem because important identity-related facial regions may be hidden or distorted [2]. Occlusions can be caused by masks, sunglasses, scarves, medical coverings, or other occluding objects. This issue became especially visible during the recent pandemic, when the use of face masks was mandatory, but it is not limited to pandemic-related scenarios. In practical recognition systems, partial occlusion can occur in public spaces, workplaces, healthcare environments, educational institutions, and surveillance settings. Therefore, robust recognition of partially occluded faces is essential for reliable camera-based biometric sensing systems, especially when multiple faces must be detected and recognized simultaneously in real time.

Face recognition under occlusion using deep learning models, CNN-based feature extractors, and classifier-based decision stages has been addressed by recent papers. CNN architectures have been effective in learning identity-relevant facial features, while conventional classifiers such as Support Vector Machines (SVMs) have also been used for final identity classification in hybrid pipelines. Previous studies have shown that CNN-based feature extraction and hybrid CNN-with-SVM classification strategies can achieve competitive performance in face recognition tasks [3,4]. However, many existing works differ in terms of detector, preprocessing procedure, feature extractor, classifier, dataset, and evaluation protocol. As a result, it is often difficult to determine whether performance improvements are caused by the feature extraction model, the face detector, the classifier, or the dataset conditions.

This limitation is particularly important in real-time and edge-device applications. In such settings, accuracy alone is not sufficient to evaluate the practical value of a recognition pipeline. A system may achieve high offline accuracy but still be unsuitable for deployment if its inference speed is low, latency is high, or computational requirements exceed the capabilities of the target device. Therefore, robust face recognition systems intended for edge deployment should be evaluated using both offline recognition metrics and real-time performance indicators such as frames per second (FPS), per-frame latency, and device-level feasibility.

The need for robust real-time visual understanding is not limited to face recognition. Chen et al. [5] demonstrate that real-time visual understanding can support activity classification in dynamic assembly environments by combining RGB information, hand-skeleton information, and temporal activity modeling. Their study shows that reliable perception is important when visual systems must operate under changing and practical conditions. Similarly, real-time face recognition systems deployed on edge devices should be evaluated not only in terms of recognition accuracy, but also with respect to latency, deployment feasibility, and robustness under partial occlusion, pose variation, illumination changes, and computational constraints.

Another important limitation in previous evaluation protocols is the use of private or custom datasets only. Although custom datasets are useful for controlled experimentation, evaluation on public benchmark datasets is necessary to improve comparability and generalizability. Public occluded face datasets allow researchers to assess whether a proposed method remains effective beyond the conditions under which the custom dataset was collected. Therefore, this study evaluates the proposed pipelines on both a custom dataset and the public Real-World Occluded Faces (ROF) dataset [2]. The use of the ROF dataset provides an additional validation scenario involving neutral, sunglasses, and masked-face conditions.

To address these limitations, this study presents a controlled evaluation of three hybrid multi-face recognition pipelines for real-time partially occluded face recognition. In contrast to comparisons where several components vary at the same time, the proposed evaluation keeps the face detector, preprocessing protocol, classifier, unknown rejection threshold, and evaluation procedure fixed across all pipelines. Specifically, all pipelines use the same SCRFD face detector [6], the same preprocessing procedure, the same Linear SVM classifier, and the same 60% unknown rejection threshold. Furthermore, to ensure a fair comparison, all pipelines were evaluated using identical image crops, preprocessing settings, and train/test partitions. The only component that varies across all of them is the feature extraction stage.

The selected feature extraction strategies represent different model families and design motivations. ResNet29 is used as a compact residual CNN-based feature extraction baseline. ConvNeXt is included as a modern convolutional architecture inspired by recent advances in deep visual representation learning [7]. ResNet100 with ArcFace embeddings is commonly used as a face-recognition-specific embedding model designed to produce highly discriminative facial embeddings [8]. By evaluating these feature extractors under the same detection, preprocessing, SVM classification, and thresholding conditions, the study aims to isolate the influence of the feature extraction stage on recognition performance under partial occlusion.

The use of a linear SVM classifier in this study is motivated by its computational efficiency, suitability for small- and medium-sized embedding datasets, and practical value in edge-oriented recognition systems. Unlike end-to-end SoftMax-based CNN classification, the SVM-based design separates feature extraction from identity classification. This separation allows the classifier to be retrained more easily when identities are added or removed, without requiring the full retraining of the deep feature extraction model. In this work, the SVM is not presented as the main source of novelty; rather, it is used as a fixed and lightweight classification component to ensure that the comparison focuses on the effect of the feature extractor.

The evaluation protocol is designed to reduce data leakage and better reflect real-world occlusion scenarios. The models are trained only on normal, non-occluded face images and tested on unseen partially occluded face images. This setup evaluates whether facial representations learned from non-occluded images can generalize to occluded conditions such as sunglasses and masks. Performance is measured using confusion-matrix-based accuracy, precision, recall, F1 score, ROC/AUC, and equal error rate (EER). In addition, real-time deployment performance is evaluated separately using FPS and latency measurements on a GPU-enabled laptop (ASUS X705UDR, ASUSTeK Computer Inc., Taipei, Taiwan) and a Raspberry Pi 4 (Raspberry Pi 4 Model B, 8 GB RAM, Raspberry Pi Ltd., Cambridge, UK; manufactured in China). The implementation was developed in Visual Studio Code (v1.124.2) and executed using Python (v3.11.0) on the GPU-enabled laptop and Python (v3.10) on the Raspberry Pi 4.

In addition to evaluating the proposed pipelines, this study also includes a comparison with three existing occluded face recognition methods for which public code is available [9,10,11]. These methods are evaluated on the custom dataset using both offline quantitative metrics and real-time recognition testing. For the public Real-World Occluded Faces dataset, the comparison is limited to offline evaluation, including confusion matrices, accuracy, precision, recall, F1-score, ROC/AUC, and Equal Error Rate (EER). This separation is adopted because the custom dataset is used for practical real-time deployment analysis, whereas the public dataset is used as a benchmark for reproducible offline evaluation under occlusion.

The main contributions of this study are summarized as follows:

1. A controlled hybrid multi-face recognition framework for partial occlusion analysis is proposed, comparing ResNet29, ConvNeXt, and ResNet100 with ArcFace embeddings under the same image crops, preprocessing procedures, train/test splits, linear SVM classifier, unknown rejection threshold, and evaluation protocol. In this setting, only the feature extraction module differs, while all models are trained on normal, non-occluded faces and tested on unseen partially occluded faces.
2. A harmonized comparison with three existing occluded face recognition methods with publicly available code is conducted across the custom dataset and public benchmark datasets. All methods are evaluated using consistent face-cropping, preprocessing, and evaluation procedures to support a fairer comparison across pipelines and datasets.
3. A unified offline, real-time, and edge-device evaluation is provided, combining recognition metrics such as accuracy, precision, recall, F1-score, ROC/AUC, and EER with deployment metrics such as FPS and latency on a GPU-enabled laptop and Raspberry Pi 4.

This paper addresses the following research questions based on the paper’s contributions:

1. How does a Convolutional Neural Network (CNN) for feature extraction affect the recognition performance in a controlled multi-face recognition pipeline on partially occluded faces?
2. To what extent can a hybrid pipeline based on SCRFD face detection, deep feature extraction, and Linear SVM classification generalize from normal, non-occluded training faces to unseen partially occluded test faces?
3. How does the best-performing pipeline behave in real-time deployment scenarios on a GPU-enabled laptop and Raspberry Pi 4 in terms of FPS and latency?

The remainder of this paper is organized as follows. Section 2 reviews related work on occluded face recognition, hybrid CNN-based recognition systems, and real-time face recognition methods. Section 3 presents the proposed methodology, including dataset preparation, face detection, preprocessing, feature extraction, SVM classification, thresholding, and the experimental protocol. Section 4 reports the offline quantitative results and real-time deployment results. Section 5 presents the main findings, limitations, comparison with existing methods, and practical suitability for edge-device deployment. Finally, Section 6 concludes the paper and outlines future work directions.

## 2. Related Work

Partially occluded face recognition remains a challenging problem because the standard recognition pipeline, including face detection, alignment, feature embedding, and matching or classification, can degrade when important discriminative facial regions are missing. Occlusions may be caused by masks, sunglasses, scarves, hats, hands, hair, bandages, or other objects covering part of the face. In addition, unconstrained acquisition conditions such as pose variation, blur, illumination changes, low resolution, and cluttered backgrounds further increase the difficulty of reliable recognition.

A major direction in occluded face recognition focuses on learning more robust facial embeddings from the visible regions of the face. Several studies have proposed attention-based or region-aware models that assign more importance to visible identity cues, especially the upper facial region when the mouth and nose are occluded. For example, Wang et al. [12] proposed a lightweight dual-branch convolutional self-attention network that combines global and partial facial cues and increases the contribution of upper-face information. Other works have explored new backbones and training strategies to reduce intra-class variation caused by occlusion and to improve masked-face recognition performance [13]. Kumar et al. [9] evaluated multiple CNN backbones and introduced a modified MobileNetV2-based design using transfer learning and upper-face-focused feature extraction, while also providing public code and dataset resources.

Another group of methods formulates occluded face recognition as a masked-unmasked matching or pairwise similarity problem. These approaches aim to compare partially visible faces with non-occluded gallery images and maintain identity consistency across different visibility conditions. Siamese-style learning has been used to learn similarity between masked and unmasked face pairs, especially when the visible region is restricted to the eyes and forehead [14,15]. Related studies also use visible-region selection, attention mechanisms, or angular-margin optimization to improve discriminative learning under partial visibility [16,17].

Feature fusion and reconstruction-based methods represent another important direction. Fusion-based methods use information from more than one source to make the system more robust against different types of occlusion, such as irregular accessories and partial coverings [18]. Reconstruction-based methods attempt to recover the missing facial region before recognition. For example, GAN-based and diffusion-based methods have been proposed to generate identity-related reconstructions of occluded faces, allowing the recognition model to exploit information from completed facial features [19,20]. Although these methods can improve recognition under severe or irregular occlusion, they often introduce additional computational cost, which may make these methods less practical for real-time recognition and edge-device deployment, particularly in applications such as mobile devices or surveillance systems where processing power is constrained.

Face detection is also a critical component in occluded face recognition. When the face is heavily or irregularly covered, detection and localization may fail before the recognition stage begins. Poor cropping and alignment can reduce embedding quality and directly affect classification performance. Alashbi et al. [21] highlighted this issue by proposing an occluded face detection methodology and a dataset designed for occlusion-heavy conditions. This shows that robust detection is essential for practical systems, especially when the goal is to recognize multiple people and display identity labels in real time.

In parallel, lightweight and real-time recognition methods have been investigated for deployment in constrained environments such as attendance systems, surveillance, embedded devices, and IoT applications. These settings require not only high recognition accuracy but also low latency, low computational cost, and stable performance under real-world capture conditions. Ma et al. [22] proposed a lightweight face recognition network and reported robustness under partial occlusions such as sunglasses and scarves, together with practical efficiency measurements. Other studies have considered secure and decentralized recognition for IoT using deep features with lightweight classification components [23], efficient recognition under challenging drone imagery [24], and real-time recognition systems where throughput, latency, and prediction stability are important factors [10,25].

Despite these advances, fair comparison across occluded face recognition methods remains difficult. Many studies use different datasets, detectors, preprocessing procedures, feature extractors, classifiers, and evaluation protocols. Some works focus mainly on generated or fixed mask occlusions, while others evaluate under different real-world occlusion conditions [16,17]. These differences make it difficult to determine whether performance improvements are caused by the recognition model itself or by differences in dataset conditions, detection quality, preprocessing, or evaluation setup.

Table 1 highlights the alignment of each publication with the objectives established in this study and offers a concise evaluation of the degree to which principal issues in occluded face recognition are tackled.

As summarized in Table 1, previous studies have addressed important aspects of occluded face recognition, including masked-face recognition, feature learning, visible-region attention, reconstruction, and lightweight deployment. However, fewer works provide a controlled evaluation in which multiple hybrid pipelines are compared under the same detector, preprocessing protocol, classifier, thresholding strategy, dataset conditions, and evaluation metrics. This study addresses this gap by comparing ResNet29 with linear SVM, ConvNeXt with linear SVM, and ResNet100 with ArcFace embeddings with linear SVM under a unified experimental protocol. In addition, three existing public-code methods [9,10,11] are evaluated under the same available testing conditions where applicable, allowing a more transparent comparison with recent approaches.

## 3. Materials and Methods

This section describes the materials and methods used to evaluate the proposed hybrid multi-face recognition pipelines under partial occlusion. The study was designed as a controlled comparison in which the face detector, preprocessing strategy, classifier, unknown rejection threshold, and evaluation metrics were kept fixed across the proposed pipelines. The only component varied among the three proposed pipelines was the feature extraction stage.

### 3.1. System Workflow

**Figure 1:**
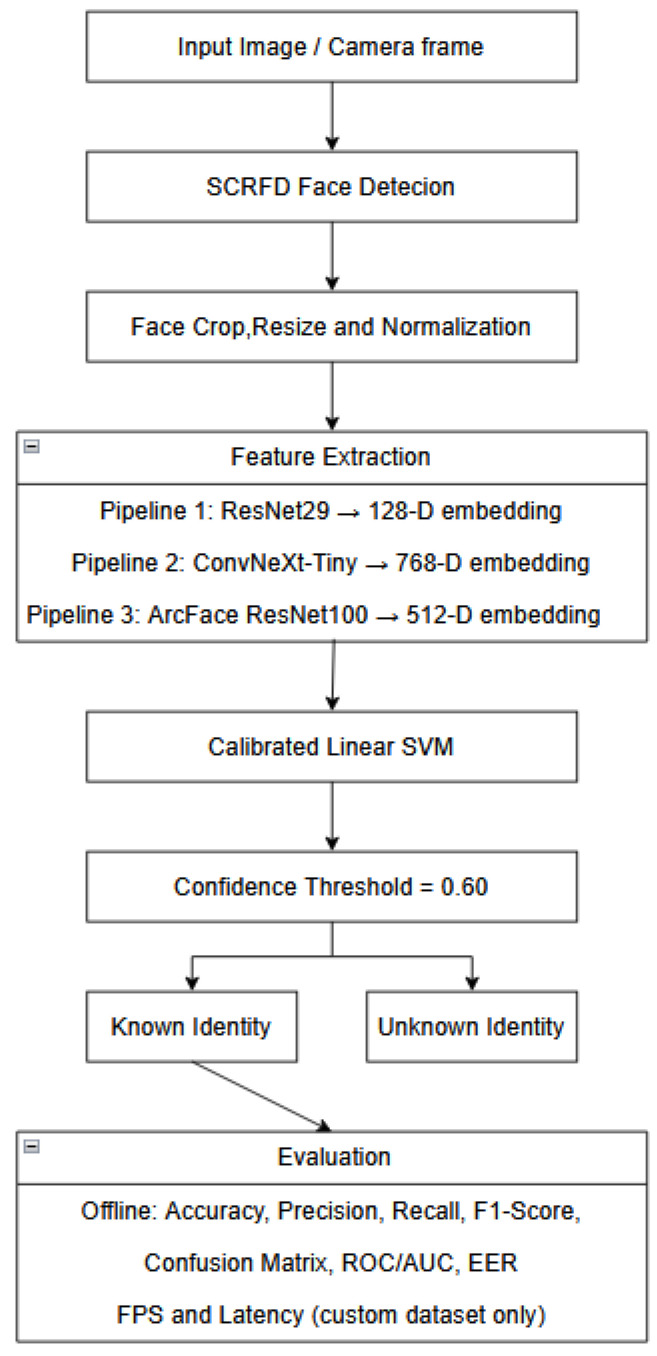
Flow diagram of the proposed controlled multi-face recognition pipeline.

Figure 1 presents the general workflow of the proposed system consists of dataset preparation, face detection, preprocessing, feature extraction, classification, and evaluation. First, facial images were collected from the custom dataset or selected from the public Real-World Occluded Faces (ROF) dataset. Second, the SCRFD detector was used to localize the facial region. Third, the detected face crop was resized and normalized according to the input requirements of the corresponding feature extractor. Finally, the extracted embeddings were classified using a calibrated Linear SVM with a fixed confidence threshold for unknown rejection. The SVM hyperparameters and the unknown rejection threshold were selected using a separate validation subset composed of non-occluded facial images. This validation subset was independent of the training set and was used only for model selection, confidence-score analysis, and selection of the fixed threshold of 0.60. No images from the final custom occlusion test set or from the ROF sunglasses and masked-face test subsets were used during hyperparameter tuning, threshold selection, or calibration. Therefore, the final reported results were obtained on test data that remained fully separated from training, validation, and threshold selection.

The experiments were divided into offline quantitative evaluation and real-time recognition testing. Training, feature extraction, parameter selection, and offline evaluation were performed on a GPU-enabled laptop. Offline evaluation was conducted on both the custom dataset and the ROF dataset, whereas real-time recognition testing was performed only on the custom dataset using live-camera input. The real-time experiments were conducted on the same GPU-enabled laptop and on a Raspberry Pi 4 Model B to assess deployment feasibility under different computational conditions. Since the Raspberry Pi 4 does not include an integrated camera, an external USB camera was used for real-time image acquisition.

### 3.2. Hardware Environment

Two devices were used in this study: a GPU-enabled laptop and a Raspberry Pi 4 Model B. The GPU-enabled laptop’s main specifications are presented in Table 2; this laptop was used for the training phase, feature extraction, parameter selection, offline evaluation, and real-time testing. After training, the trained classifier models and required pipeline components were transferred to the Raspberry Pi 4 model. The Raspberry Pi 4 Model B was used to evaluate the feasibility of deploying the trained recognition pipelines on a resource-constrained edge device.

Table 3 describes the Raspberry Pi 4 Model B specifications used for edge-device real-time testing.

After the models were trained on the GPU-enabled laptop, the Raspberry Pi 4 Model B was used only for real-time deployment evaluation. This allowed the system to be tested on two devices.

For live image capture, an external USB webcam was used because the Raspberry Pi 4 does not have its own camera. The same webcam was used for both devices.

To ensure the same frame capture conditions during real-time testing, both devices used the webcam described in Table 4. The reported FPS and latency values demonstrate the practical usability of the system on both devices.

### 3.3. Dataset Preparation

Informed consent was obtained before data collection, including permission to use the collected facial images for academic research and publication purposes only in anonymized or blurred form. For participants under the age of 18, consent was obtained from a parent, since the dataset includes family members and friends, with ages ranging from 7 to 40 years. No identifiable facial images are published in the manuscript, and any visual examples included in the paper are blurred to prevent participant identification.

During the initial custom dataset acquisition stage, Haar Cascade was used only as an automatic face-localization tool to assist frame capture and face-region extraction from the camera stream. However, Haar Cascade was not used in the final recognition pipelines or in the controlled evaluation. For all final experiments, SCRFD was used as the common face detector across the proposed pipelines to avoid detector-related bias. Informed consent was obtained before data collection. The images were anonymized and used only for academic research purposes, and no additional biometric or sensitive personal information was stored beyond the facial images required for the experiments.

To reduce data leakage and evaluate robustness under realistic occlusion conditions, only normal, non-occluded face images were used for training and validation. The partially occluded images were reserved for testing. Therefore, the same identities appear in training and testing, but the training set contains normal faces, whereas the testing set contains occluded face conditions. This protocol evaluates whether feature representations learned from non-occluded faces can generalize to unseen occluded conditions.

Table 5 presents the number of training and testing images used for each subject after preprocessing. A validation subset was initially created from the normal-face training images using an 80/20 split. This validation subset was used for parameter selection and unknown rejection threshold tuning. After selecting the final classifier configuration and threshold, the validation images were merged back into the training set, and the final classifier was trained using all available normal-face training images.

In addition to the custom dataset, the public ROF dataset was used to evaluate the proposed pipelines under public benchmark conditions. The ROF dataset contains neutral, sunglasses, and masked-face subsets. In this study, the neutral subset was used for training and validation, while the sunglasses and masked subsets were used for testing. For the sunglasses evaluation, 47 identities were selected by retaining only subjects with at least 30 neutral images for training and at least 5 sunglasses images for testing. For the masked-face evaluation, 19 identities were selected based on the availability of at least 20 neutral images for training and at least 4 masked images for testing. The same identities were used across the neutral and occluded subsets for each ROF evaluation condition. Similar to the custom dataset protocol, the ROF validation subset was used for parameter and threshold selection, after which the training and validation images were combined and the final model was tested separately on the sunglasses and masked subsets.

### 3.4. Face Detection and Preprocessing

All proposed pipelines used SCRFD as the face detector to ensure consistent face localization and avoid detector-related bias. Therefore, performance differences can be mainly attributed to the feature extraction stage rather than variations in detection quality.

After detection, the facial region was cropped and resized according to the input requirements of the corresponding feature extractor. The same general preprocessing strategy was used across the proposed pipelines: SCRFD-based face detection, face cropping, resizing, conversion to floating-point representation, and normalization before feature extraction. No separate landmark-based facial alignment step was implemented.

For the ArcFace ResNet100 pipeline, the standard ArcFace-style normalization was used:

$$x^{\prime }=\frac{x-127.5}{128}$$

where *x* is the original pixel value and $x^{\prime }$ is the normalized value. For the ResNet29 and ConvNeXt pipelines, the detected face crops were normalized according to the input requirements of the corresponding pretrained feature extractor.

### 3.5. Feature Extraction Pipelines

Three hybrid feature extraction pipelines were evaluated. In all cases, the original classification heads of the deep models were not used for identity prediction. Instead, the deep models were used as frozen feature extractors, and the extracted embeddings were passed to a Linear SVM classifier.

The first pipeline uses SCRFD for face detection and ResNet29 as the feature extractor. ResNet29 produces a 128-dimensional embedding for each detected face. These embeddings are then used as input to the Linear SVM classifier.

The second pipeline uses SCRFD for face detection and ConvNeXt-Tiny as the feature extractor. ConvNeXt-Tiny produces a 768-dimensional representation for each face image. This representation is used as the input feature vector for the same Linear SVM classification stage.

The third pipeline uses SCRFD for face detection and a pretrained ArcFace ResNet100 model for feature extraction. This model produces 512-dimensional face embeddings. These embeddings are then classified using the same calibrated Linear SVM classifier. The ArcFace ResNet100 pipeline represents the face-recognition-specific embedding configuration in this study.

This design separates feature extraction from classification. It also allows the classifier to be retrained when identities are added or removed, without requiring full retraining of the deep feature extraction model. Since the detector, preprocessing strategy, classifier, and thresholding rule are fixed, the comparison focuses on the effect of the feature representation.

### 3.6. Classification with Linear SVM and Unknown Rejection

The classification stage was performed using a Linear SVM. The same classifier type was used for all proposed pipelines in order to maintain a controlled comparison. For the ArcFace ResNet100 pipeline, the 512-dimensional embeddings were classified using a calibrated Linear SVM. The base classifier was implemented using LinearSVC with C=1.0, balanced class weights, an L2 penalty, squared-hinge loss, and a one-vs-rest multi-class strategy.

Because LinearSVC does not directly provide probability estimates, sigmoid calibration with 5-fold cross-validation was applied. The calibrated confidence scores were then used for unknown rejection. A detected face was assigned to the predicted identity only if the maximum calibrated SVM confidence was at least 0.60. If the maximum confidence was below 0.60, the sample was labeled as unknown. This threshold was selected using the validation subset and then fixed for final testing.

The unknown rejection mechanism is important for practical real-time recognition systems because the system may encounter individuals who are not included in the training set. Therefore, the classifier should not be forced to assign every detected face to one of the known identities.

### 3.7. Evaluation Protocol

The evaluation was performed in two parts: offline quantitative evaluation and real-time recognition testing. Offline evaluation was used to measure recognition performance on complete test sets, while real-time testing was used to assess live-camera deployment behavior, FPS, and latency.

On the custom dataset, the models were trained using non-occluded face images and tested on unseen partially occluded face images, including masked faces, left-eye patches, right-eye patches, and nasal patches. For the ROF dataset, the models were trained using neutral images and tested separately on the sunglasses and masked subsets. Real-time testing was not performed on the ROF dataset; it was used only for offline benchmark evaluation.

The offline metrics included accuracy, precision, recall, F1-score, confusion matrices, ROC/AUC, and Equal Error Rate (EER). Precision, recall, and F1-score were reported using weighted averages to account for class imbalance across identities. Confusion matrices were used to examine class-specific recognition behavior and identify misclassification patterns. ROC/AUC and EER were calculated using genuine and impostor score distributions. Genuine scores correspond to comparisons involving the same identity, whereas impostor scores correspond to comparisons involving different identities. EER was obtained at the operating point where the false acceptance rate and false rejection rate were approximately equal.

In addition to the proposed pipelines, three existing methods with publicly available code were included as comparison baselines [9,10,11]. The original implementation logic of each method was preserved as much as possible, while the training and testing data were adapted to the custom and ROF dataset protocols used in this study. These methods were retrained and evaluated on the same testing subsets as the proposed pipelines. On the custom dataset, they were assessed using both offline quantitative metrics and real-time recognition testing. On the ROF dataset, they were assessed using offline benchmark metrics only. The detailed detector, feature extractor, classifier, and performance results for these methods are presented in the Section 4.

Real-time recognition testing was conducted on the custom dataset using live USB-camera input. The trained pipelines were integrated into a live recognition interface that detects faces, predicts identity labels, displays confidence scores, and reports FPS and latency. A multi-face real-time test was performed using five visible faces in a single frame to verify simultaneous recognition capability. FPS and latency were measured using a single-face live-camera scenario over continuous live-camera execution. The reported values correspond to the final average FPS and average latency after processing thousands of frames for each method.

The real-time results were interpreted at the full pipeline implementation level. This is important because speed is affected not only by the depth of the neural network but also by the runtime backend, hardware acceleration support, and implementation optimization. For example, the ResNet29-based pipeline was affected by the limited GPU optimization of the pretrained model used in this configuration. In contrast, the other pipelines were tested using GPU-supported execution where available.

## 4. Results

This section presents the experimental results of the proposed hybrid multi-face recognition pipelines and the selected public-code comparison methods. The evaluation is divided into three parts. First, offline quantitative results are reported on the custom partially occluded face dataset. Second, real-time recognition performance is evaluated on the custom dataset using live-camera input. Third, offline benchmark results are reported on the public Real-World Occluded Faces (ROF) dataset for sunglasses and masked-face testing. The results are reported using accuracy, precision, recall, F1-score, confusion matrices, ROC/AUC, Equal Error Rate (EER), FPS, and latency.

### 4.1. Offline Evaluation on the Custom Dataset

Table 6 presents the offline quantitative results on the custom dataset. In this evaluation, the models were trained using normal, non-occluded face images and tested on unseen partially occluded images, including masked faces, left-eye patches, right-eye patches, and nose patches. Results are reported for both closed-set evaluation and threshold-based evaluation using the 0.60 unknown rejection threshold.

As shown in Table 6, the SCRFD + ArcFace ResNet100 + Linear SVM pipeline achieved the best performance on the custom dataset, reaching 99.55% closed-set accuracy and 99.20% threshold-based accuracy. The ROC/AUC analysis produced an AUC of 99.33% and an EER of 0.0092, indicating strong separation between genuine and impostor score distributions. The ResNet29-based pipeline also achieved strong performance, while the ConvNeXt-based pipeline showed lower recognition performance, particularly under the threshold-based setting.

The public-code comparison methods showed lower performance on the custom dataset under the adopted protocol. The Kumar et al. [9] method showed the weakest transferability, which may be related to the difference between its original masked-face recognition setting and the broader partial occlusion conditions used in this study. The Alansari et al. [10] and Li et al. [11] methods performed better than Kumar et al. [9], but remained below the best proposed pipeline.

**Figure 2:**
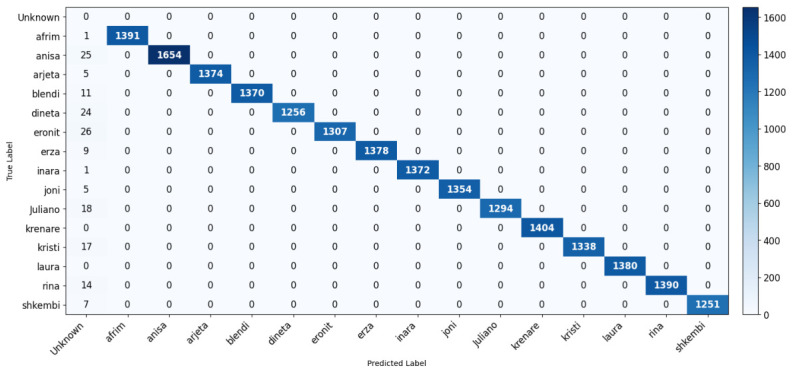
Confusion matrix of the SCRFD + ArcFace ResNet100 + Linear SVM pipeline on the custom partially occluded face dataset. Most samples are correctly classified along the diagonal, while the remaining errors are mainly rejected as unknown.

Figure 2 presents the threshold-based confusion matrix of the best-performing pipeline on the custom dataset. The matrix shows strong diagonal dominance, confirming that most identities were correctly recognized under partial occlusion. The ‘Unknown’ class represents samples rejected by the confidence-based threshold instead of being forced into an incorrect identity class.

### 4.2. Open-Set Unknown-Identity Evaluation

Table 7 reports the open-set unknown-identity rejection results using 1100 images from identities that were not included during training. This experiment directly addresses the open-set scenario by evaluating whether each pipeline can reject novel identities rather than forcing them into one of the enrolled classes. At the selected operating threshold of 0.60, the ArcFace ResNet100 pipeline correctly rejected 1052 out of 1100 unknown images, corresponding to a 95.64% unknown rejection rate and a 4.36% false acceptance rate. The ConvNeXt-Tiny and ResNet29 pipelines achieved unknown rejection rates of 90.64% and 87.55%, respectively, at the same threshold. Li et al. [11] also achieved very strong unknown rejection, with 99.91% at threshold 0.60, while Kumar et al. [9] and Alansari et al. [10] rejected all unknown identities across the evaluated thresholds. However, these results should be interpreted together with the known-identity recognition results, since perfect rejection of unknown identities alone does not necessarily indicate the best overall recognition performance if the method also rejects or misclassifies valid enrolled identities.

### 4.3. Real-Time Recognition Results on the Custom Dataset

Real-time recognition testing was performed only on the custom dataset using live-camera input. The purpose of this experiment was to evaluate practical deployment behavior, including FPS and latency. Table 8 reports the real-time performance measured during continuous live-camera execution. The number of processed frames is included to show that FPS and latency were measured over thousands of frames.

**Figure 3:**
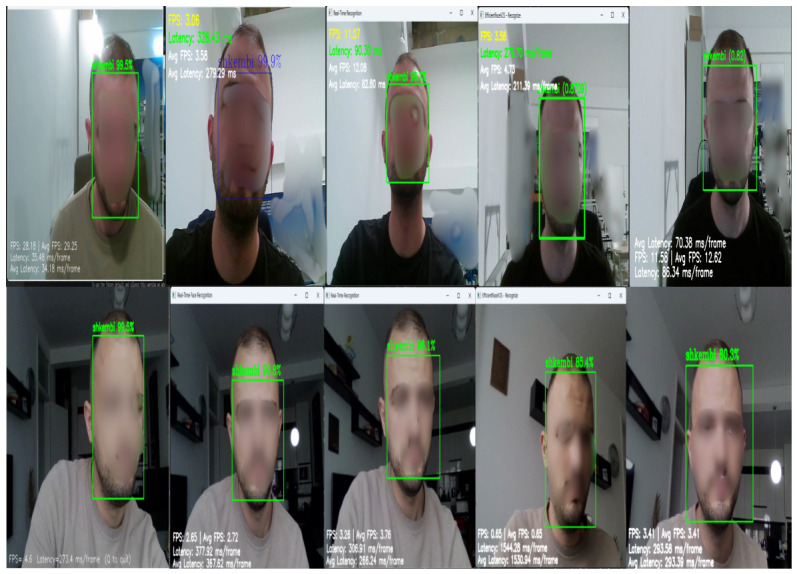
Representative real-time recognition outputs on the custom dataset. The upper row shows results obtained on the GPU-enabled laptop, while the lower row shows results obtained on the Raspberry Pi 4. From left to right, the columns show: SCRFD + ArcFace ResNet100 + Linear SVM, SCRFD + ResNet29 + Linear SVM, SCRFD + ConvNeXt-Tiny + Linear SVM, Alansari et al. [10], and Li et al. [11]. The examples illustrate live-camera FPS and latency behavior across the proposed pipelines and selected public-code comparison methods. For clarity, the corresponding real-time performance values are reported in Table 8.

Table 8 summarizes the numerical FPS and latency results, while Figure 3 provides representative live-camera outputs from the same real-time experiments.

### 4.4. Real-Time Performance on Edge Devices

To complement the FPS and latency measurements, Table 9 reports the runtime-resource profile of the evaluated pipelines on the Raspberry Pi 4 Model B, including monitored CPU usage, monitored RAM consumption, estimated power usage, and overall edge-runtime demand.

CPU usage and RAM consumption were monitored during real-time face recognition on the Raspberry Pi 4 Model B, while power usage was estimated based on the observed runtime behavior, including FPS, latency, and relative computational demand. Therefore, the power values should be interpreted as approximate deployment indicators rather than direct power-meter measurements.

The SCRFD + ArcFace ResNet100 + Linear SVM pipeline achieved the highest real-time performance on the GPU-enabled laptop, reaching 29.08 FPS with an average latency of 34.39 ms/frame. On the Raspberry Pi 4, the same pipeline achieved 4.60 FPS with an average latency of 273.40 ms/frame. Although the Raspberry Pi performance was lower than the GPU-enabled laptop performance, the result demonstrates the feasibility of deploying the proposed pipeline on an edge device. Among the tested Raspberry Pi configurations, ArcFace ResNet100 and ConvNeXt-Tiny showed the strongest FPS values, while the Alansari et al. [10] method was the slowest due to higher processing latency.

The ResNet29-based pipeline achieved lower real-time speed. This result should be interpreted at the full pipeline-implementation level because FPS is affected not only by network depth but also by the runtime backend, hardware acceleration support, and model optimization. In this implementation, the pretrained ResNet29 model was affected by limited GPU optimization, whereas the ArcFace ResNet100 pipeline benefited from a more optimized runtime.

**Figure 4:**
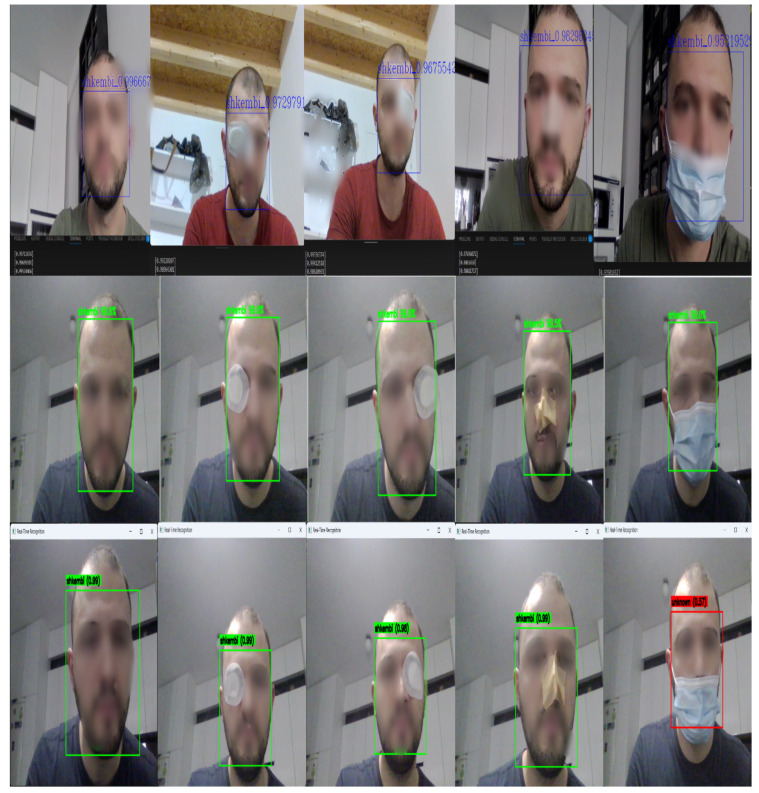
Representative real-time recognition examples of the three proposed pipelines under different custom-dataset occlusion conditions. From top to bottom: SCRFD + ResNet29 + Linear SVM, SCRFD + ArcFace ResNet100 + Linear SVM, and SCRFD + ConvNeXt-Tiny + Linear SVM. From left to right, the columns show normal face, left-eye occlusion, right-eye occlusion, nose occlusion, and mask occlusion. The displayed values correspond to classifier confidence scores for the predicted identities. For clarity, the corresponding average values and quantitative results for this figure are reported in Table 10.

Figure 4 presents representative real-time recognition examples for the three proposed pipelines under different custom-dataset occlusion conditions. Each row corresponds to one pipeline: the first row shows SCRFD + ResNet29 + Linear SVM, the second row shows SCRFD + ArcFace ResNet100 + Linear SVM, and the third row shows SCRFD + ConvNeXt-Tiny + Linear SVM. The columns show the same sequence of occlusion types. The displayed values above the detected faces correspond to classifier confidence scores for the predicted identity. These qualitative examples illustrate live-camera behavior, while the quantitative performance is reported in Table 6 and Table 8.

**Figure 5:**
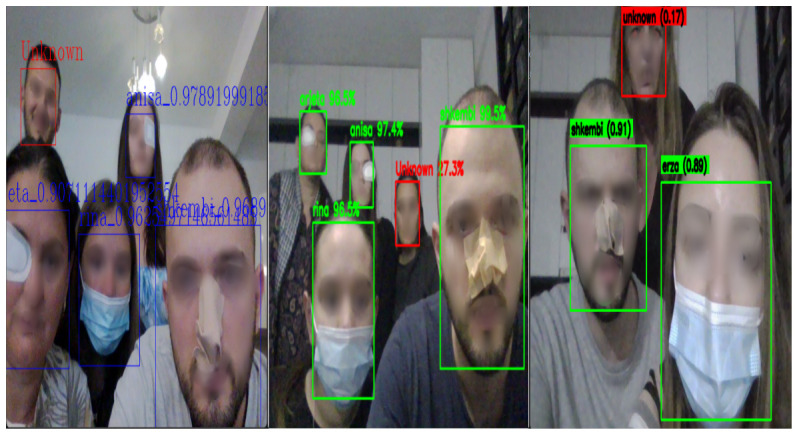
Multi-face real-time recognition examples using partially occluded faces in a single frame. From left to right: SCRFD + ResNet29 + Linear SVM, SCRFD + ArcFace ResNet100 + Linear SVM, and SCRFD + ConvNeXt-Tiny + Linear SVM. The displayed values correspond to classifier confidence scores for the predicted identity of each detected face. For clarity, the corresponding average confidence scores are reported in Table 10.

Figure 5 shows a multi-face real-time recognition example. The first image corresponds to SCRFD + ResNet29 + Linear SVM, the second image corresponds to SCRFD + ArcFace ResNet100 + Linear SVM, and the third image corresponds to SCRFD + ConvNeXt-Tiny + Linear SVM. This experiment demonstrates the ability of the live system to detect and label multiple partially occluded faces in the same frame. The displayed values above the bounding boxes represent classifier confidence scores for the predicted identities and are also used for confidence-based unknown rejection.

Table 10 reports representative confidence scores observed during live-camera testing. These values correspond to displayed prediction confidence scores.

### 4.5. Qualitative Difficult Cases

During real-time testing, several challenging conditions were observed, including aging-related appearance variation, most of the face occluded, an inclined face angle, and insufficient illumination. These cases were used as qualitative observations to examine the behavior of the proposed pipelines under practical live-camera conditions.

**Figure 6:**
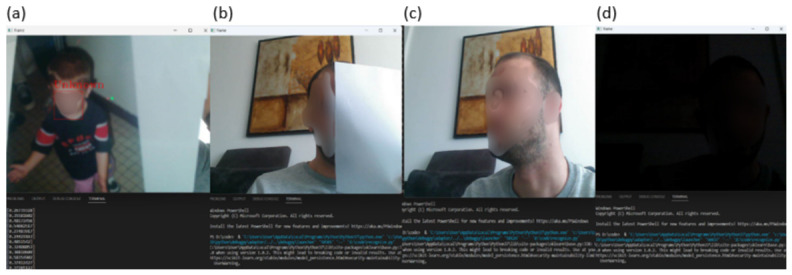
Representative difficult cases observed during real-time testing: (**a**) aging-related appearance variation, (**b**) most of the face occluded, (**c**) inclined/side face angle, and (**d**) insufficient illumination.

The examples in Figure 6 show conditions where recognition becomes more difficult. In particular, the ResNet29-based and ConvNeXt-based pipelines were more affected by inclined face angle, insufficient illumination, and severe object-based occlusion during qualitative live-camera testing. These conditions reduced detection quality or classification confidence and made recognition less stable.

**Figure 7:**
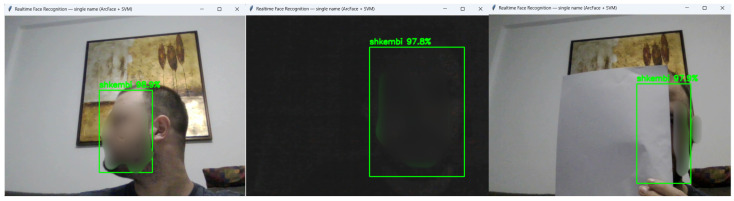
Challenging real-time examples in which the SCRFD + ArcFace ResNet100 + Linear SVM pipeline still produced high confidence scores under inclined face angle, low illumination, and most of the face occluded. The displayed values correspond to classifier confidence scores for the predicted identity.

As shown in Figure 7, the SCRFD + ArcFace ResNet100 + Linear SVM pipeline handled these difficult real-time conditions more effectively than the ResNet29-based and ConvNeXt-based pipelines. In the tested examples, the ArcFace-based pipeline still produced high confidence scores under inclined face angle, low illumination, and most of the face occluded, while the other two proposed pipelines did not show the same level of stability in these cases.

However, aging-related appearance variation remained a limitation. This issue occurs when the subject’s current facial appearance differs noticeably from the training images. Therefore, periodic dataset updates or retraining may be required when a subject’s facial appearance changes significantly.

Overall, these qualitative examples show that partial occlusion is not the only challenge in real-time face recognition. Face scale, pose, illumination, severe object-based occlusion, and aging-related appearance changes can also affect detection and classification reliability. The ArcFace-based pipeline reduced the impact of several of these real-time difficulties, but aging-related appearance variation remains an open challenge for future improvement.

### 4.6. Offline Evaluation on the ROF Sunglasses Subset

Table 11 and Table 12 present the offline benchmark results on the ROF sunglasses subset. In this evaluation, models were trained using neutral face images and tested on sunglasses images from the same identities. Closed-set results are reported separately from threshold-based results to improve readability and to distinguish forced identity classification from confidence-based unknown rejection.

The closed-set results show the performance when each test image is forced into one of the known identity classes. Under this setting, the ArcFace ResNet100 and Alansari et al. [10] methods achieved the highest closed-set performance on the sunglasses subset.

On the ROF sunglasses subset, the SCRFD + ArcFace ResNet100 + Linear SVM pipeline achieved 98.66% closed-set accuracy and 96.35% threshold-based accuracy. The threshold-based result remained high, with a low rejection rate of 3.65%. Alansari et al. [10] achieved slightly higher closed-set accuracy; however, its threshold-based accuracy decreased substantially because of a higher reject rate. This shows that closed-set accuracy alone does not fully describe recognition behavior when confidence-based rejection is applied.

**Figure 8:**
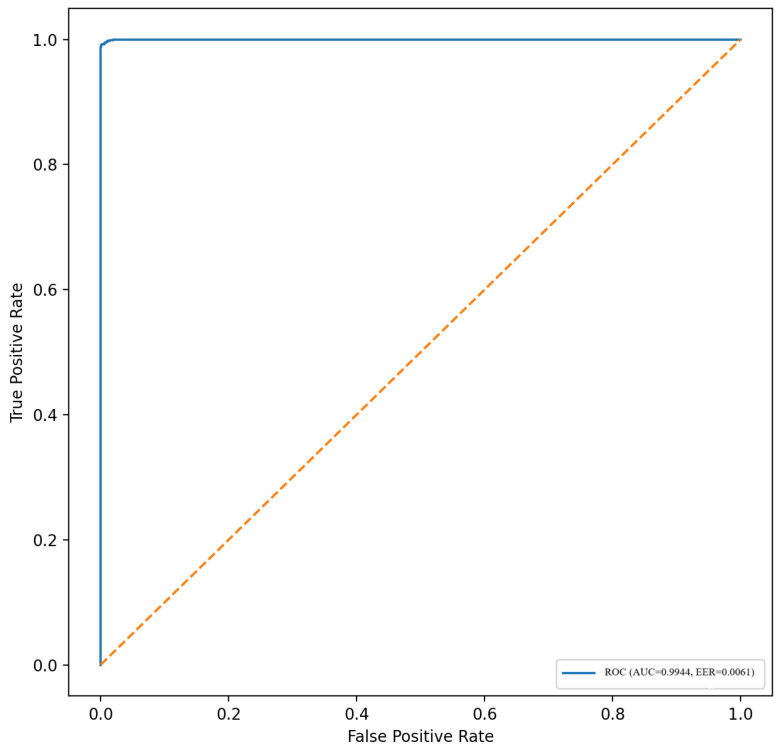
ROC curve of the SCRFD + ArcFace ResNet100 + Linear SVM pipeline on the ROF sunglasses test set, showing the corresponding AUC and EER values. The orange dashed diagonal line represents the random-classifier baseline and is included only as a reference for interpreting the ROC curve.

Figure 8 presents the ROC curve of the SCRFD + ArcFace ResNet100 + Linear SVM pipeline on the ROF sunglasses subset. The AUC and EER values indicate the verification-level separation between genuine and impostor scores under sunglasses occlusion.

### 4.7. Offline Evaluation on the ROF Masked-Face Subset

Table 13 and Table 14 present the offline benchmark results on the ROF masked-face subset. This subset is challenging because masks cover the lower facial region and reduce the amount of visible identity information. Closed-set results are reported separately from threshold-based results to improve readability and to distinguish forced identity classification from confidence-based unknown rejection.

The closed-set results show that the SCRFD + ArcFace ResNet100 + Linear SVM pipeline achieved the highest closed-set accuracy among the proposed pipelines on the ROF masked-face subset. Alansari et al. [10] also achieved high closed-set performance; however, closed-set evaluation forces each image into a known identity class and does not account for confidence-based unknown rejection.

The threshold-based results show a clearer difference between the evaluated methods. The SCRFD + ArcFace ResNet100 + Linear SVM pipeline achieved the strongest threshold-based performance, with 95.14% accuracy, 97.31% weighted F1-score, an AUC of 99.66%, and an EER of 0.0057. The low rejection rate of 4.86% indicates that only a small portion of masked-face samples was rejected as unknown. In contrast, several comparison methods experienced substantial performance degradation after thresholding because many samples were rejected as unknown.

**Figure 9:**
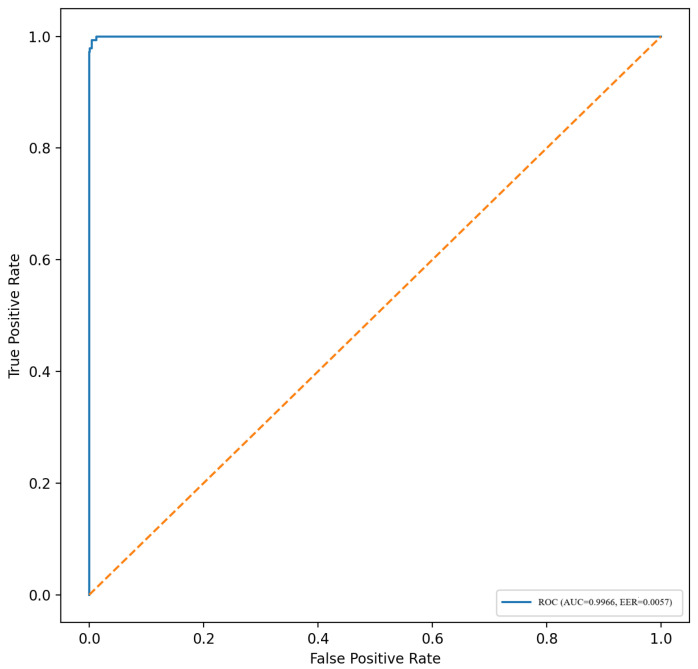
ROC curve of the SCRFD + ArcFace ResNet100 + Linear SVM pipeline on the ROF masked-face test set, showing an AUC of 0.9966 and an EER of 0.0057. The orange dashed diagonal line represents the random-classifier baseline and is included only as a reference for interpreting the ROC curve.

Figure 9 presents the ROC curve of the SCRFD + ArcFace ResNet100 + Linear SVM pipeline on the ROF masked-face subset. The AUC of 0.9966 and EER of 0.0057 confirm strong verification-level separation between genuine and impostor scores.

### 4.8. Threshold-Sensitivity Analysis

To further evaluate whether the selected unknown rejection threshold of 0.60 represents a stable operating point, an additional threshold-sensitivity analysis was performed using thresholds below and above the fixed threshold. Since the 0.60 results are already reported in the main performance tables, this analysis reports the additional thresholds 0.40, 0.50, 0.70, and 0.80. Accuracy, weighted F1-score, reject rate, and EER are reported for all proposed pipelines and selected public-code comparison methods across the custom dataset and the ROF sunglasses and masked-face subsets.

Table 15 summarizes the additional threshold-sensitivity analysis.

The additional threshold-sensitivity analysis shows how the operating threshold affects recognition and rejection behavior across datasets and methods. In general, lower thresholds increase acceptance, while higher thresholds increase the reject rate and may reduce threshold-based accuracy. This trend is particularly important for interpreting the Kumar et al. [9] results on the custom dataset. The 0.00% threshold-based accuracy obtained at the selected 0.60 threshold should not be interpreted as being caused only by the threshold setting, because the same method already achieved only 18.00% closed-set accuracy before unknown rejection was applied. The threshold-sensitivity analysis further confirms this interpretation, since Kumar et al. [9] achieved only 7.2% accuracy and 5.8% weighted F1-score even at the lower threshold of 0.40, and its performance decreased further as the threshold increased. Therefore, the low threshold-based performance mainly reflects limited transferability and domain mismatch between the method’s original design assumptions and the broader custom partial-occlusion protocol used in this study, rather than an implementation failure or a single-threshold effect. Overall, when recognition accuracy, rejection behavior, EER, and real-time deployment performance are considered together, the SCRFD + ArcFace ResNet100 + Linear SVM pipeline provided the best overall balance among the evaluated methods. The results also show that the selected 0.60 threshold was not evaluated in isolation, but was considered together with additional operating points below and above it.

### 4.9. Summary of Findings

Overall, the SCRFD + ArcFace ResNet100 + Linear SVM pipeline achieved the best balance between recognition accuracy and real-time performance among the evaluated methods. It obtained the highest custom-dataset accuracy, strong ROF performance on both sunglasses and masked-face subsets, low EER, high AUC, and the highest FPS on the GPU-enabled laptop and Raspberry Pi 4. The results also show that threshold-based evaluation is stricter than closed-set evaluation because uncertain predictions may be rejected as unknown. This distinction is important for real-time and edge-device recognition systems, where false identity assignment can be more problematic than rejecting a low-confidence sample.

The comparison with public-code methods further shows that strong performance in an original paper does not always transfer directly to a different dataset protocol or broader occlusion conditions. In particular, methods designed mainly for masked-face recognition or reconstruction-based recognition showed reduced performance when evaluated under the custom and ROF protocols used in this study. Therefore, the results support the importance of controlled evaluation using fixed detectors, classifiers, thresholds, datasets, and metrics.

## 5. Discussion

The experimental results show that the SCRFD + ArcFace ResNet100 + Linear SVM pipeline achieved the strongest overall performance among the evaluated pipelines and selected public-code comparison methods under the adopted protocol. This result can be explained by the fact that ArcFace embeddings are specifically optimized for face recognition and are designed to increase inter-class separability while reducing intra-class variation. In contrast, ConvNeXt-Tiny is a general-purpose visual backbone and showed weaker transferability to the occluded face recognition setting, especially on the ROF masked-face subset. The ResNet29-based pipeline achieved strong performance on the custom dataset and the ROF sunglasses subset, but its performance decreased under stronger mask occlusion, suggesting that the lower-dimensional 128-D representation was less robust than the 512-D ArcFace representation in more challenging occlusion scenarios. However, the superior performance of the ArcFace ResNet100 pipeline should be interpreted within the controlled experimental protocol adopted in this study. Although ArcFace embeddings are face-recognition-specific and are designed to improve identity separability, the present experiments do not fully isolate whether the observed advantage is caused only by the embedding model itself or is partly influenced by embedding dimensionality. The compared representations have different dimensions, namely 128-D for ResNet29, 512-D for ArcFace ResNet100, and 768-D for ConvNeXt-Tiny. The fact that the 768-D ConvNeXt-Tiny representation did not outperform the 512-D ArcFace representation suggests that dimensionality alone is unlikely to explain the observed performance difference. Nevertheless, a dedicated ablation study using dimensionality reduction, fixed-dimensional embeddings, and feature normalization would be required to isolate this factor more precisely.

The results also demonstrate the importance of distinguishing between closed-set and threshold-based evaluation. In the closed-set setting, each test image is forced into one of the known identity classes. This can produce high accuracy when the model is able to separate known identities, but it does not reflect the behavior of a recognition system that must reject uncertain samples. After applying the 0.60 confidence threshold, some methods showed a substantial drop in performance because many predictions were rejected as unknown. This was especially visible for some public-code comparison methods on the ROF subsets. Therefore, threshold-based evaluation provides a stricter and more realistic assessment for real-time recognition systems, where assigning a wrong identity may be more problematic than rejecting a low-confidence face.

The public-code comparison methods showed different levels of transferability under the custom and ROF protocols. Although some of these methods reported strong performance in their original studies, their performance decreased when evaluated under the broader occlusion conditions used in this work. This does not necessarily mean that the original methods are ineffective; rather, it indicates that performance can depend strongly on the dataset, occlusion type, preprocessing pipeline, threshold calibration, and original design assumptions. For example, methods designed mainly for masked-face recognition may not generalize equally well to left-eye occlusion, right-eye occlusion, nose occlusion, sunglasses, and other real-world partial occlusion cases. Similarly, reconstruction-based approaches may require careful dataset-specific calibration when transferred to different occlusion distributions. For the quantitative comparison, only methods with publicly available and executable code were included. Accordingly, three public-code methods were selected: Kumar et al. [9], Alansari et al. [10], and Li et al. [11]. These methods were adapted, reproduced, and evaluated on the custom dataset and on the ROF sunglasses and masked-face subsets using the same evaluation protocol where applicable. This selection supports reproducibility and avoids comparing the proposed pipelines only against paper-reported results obtained under different datasets or experimental settings. Several additional recent studies addressing partially occluded and masked face recognition were also reviewed and initially considered for quantitative comparison [12,13,14,15,18,19,20,21,22,24,25]. However, despite contacting the corresponding authors via email to request access to their implementations or additional reproducibility details, no usable source code was made available within the study timeline. Consequently, these methods could not be included in the quantitative experimental comparison and are instead discussed qualitatively in the Section 2.

The real-time experiments further support the practical relevance of the proposed ArcFace-based pipeline. On the GPU-enabled laptop, the SCRFD + ArcFace ResNet100 + Linear SVM pipeline achieved the highest FPS and lowest latency among the proposed pipelines. On the Raspberry Pi 4, the same pipeline achieved lower FPS, as expected, but still demonstrated edge-device feasibility. These results show that the proposed system can be used not only for offline evaluation but also for practical live-camera recognition. However, the FPS values should be interpreted at the full-pipeline implementation level, because runtime performance depends not only on model architecture but also on detector speed, preprocessing, feature extraction backend, hardware acceleration, and software optimization. The independent contribution of the SCRFD detector was not separately quantified in this study because SCRFD was intentionally kept fixed across all proposed pipelines. This design choice was made to avoid detector-related bias and to focus the controlled comparison on the feature extraction and classification stages. Therefore, the reported results should be interpreted as performance under a common SCRFD-based detection protocol, rather than as evidence that SCRFD is superior to other face detectors. Future work should include a detector-ablation study comparing SCRFD with alternative detection backbones, such as RetinaFace, YOLOv5Face, and BlazeFace, to determine whether the observed recognition trends remain consistent across different face-localization mechanisms.

The qualitative real-time examples also showed that partial occlusion is not the only difficulty in live recognition. Inclined face angle, illumination variation, most of the face covered, and aging-related appearance changes can affect recognition reliability. In the tested examples, the ArcFace-based pipeline handled inclined face angle, low illumination, and most of the face occluded more effectively than the ResNet29-based and ConvNeXt-based pipelines, producing high confidence scores in several challenging cases. However, aging-related appearance variation remained a limitation, especially when the current facial appearance differed noticeably from the training images. This suggests that periodic dataset updates or retraining may be necessary in long-term deployments.

These difficult-case observations should be interpreted as qualitative findings rather than as dedicated quantitative evaluations of pose, illumination, and temporal appearance variation. In the tested examples, the SCRFD + ArcFace ResNet100 + Linear SVM pipeline showed more stable behavior under inclined face angle, low illumination, and strong partial occlusion, producing high confidence scores in cases where the other pipelines were less stable. However, aging-related appearance variation remained a more persistent limitation when the current facial appearance differed substantially from the training images. A separate quantitative analysis of these factors would require targeted datasets or controlled acquisition protocols in which pose angle, illumination level, and temporal appearance changes are systematically varied and measured. Therefore, future work should include dedicated quantitative experiments for pose robustness, illumination robustness, and longitudinal appearance changes.

One limitation of this study is that the real-time multi-face testing was performed with a limited number of subjects in the same frame. This limitation was mainly related to camera resolution. Future work should evaluate the system with larger datasets, different camera types, wider viewing angles, and more diverse environmental conditions.

Another limitation concerns the size and representativeness of the custom dataset. Although the proposed pipelines were also evaluated on the public ROF dataset to provide additional benchmark validation under sunglasses and masked-face conditions, the custom dataset itself includes only 15 subjects collected from a limited personal context. Therefore, the custom dataset does not fully represent broader demographic diversity, age distribution, ethnic variation, or environmental variability. As a result, the findings should be interpreted as evidence of strong performance under the evaluated controlled conditions and the selected public ROF benchmark subsets, rather than as a complete demonstration of robustness across all real-world populations and deployment environments. Future work should validate the proposed pipeline on larger and more demographically diverse datasets, including broader age ranges, ethnic backgrounds, camera conditions, illumination settings, and acquisition environments.

Another important limitation is that the current system operates only on 2D facial images and does not include a liveness-detection or anti-spoofing module. Therefore, although the system can recognize partially occluded faces, it does not verify whether the detected face belongs to a live person. As a result, the system may be vulnerable to presentation attacks, such as printed photographs or screen-displayed facial images. This limitation is particularly important for security-sensitive real-world applications, where face recognition should be combined with liveness verification. Future versions of the system should integrate liveness detection, depth information, or 3D face analysis to improve resistance against spoofing attacks.

Future work will include further optimization for the Raspberry Pi, including model compression, quantization, faster detection settings, and hardware-specific inference acceleration.

## 6. Conclusions

This study presented a controlled evaluation of hybrid multi-face recognition pipelines for real-time partially occluded face recognition. The work addressed the combined problem of recognizing multiple faces while also handling partial occlusion, which is more challenging than evaluating single-face or non-occluded recognition scenarios. To support a fair comparison, the proposed pipelines used the same SCRFD face detector, preprocessing protocol, Linear SVM classifier, and 0.60 unknown rejection threshold, while varying only the feature extraction stage.

The experimental results showed that the SCRFD + ArcFace ResNet100 + Linear SVM pipeline achieved the strongest overall performance among the evaluated pipelines and selected public-code comparison methods. On the custom dataset, this pipeline achieved 99.55% closed-set accuracy and 99.20% threshold-based accuracy. On the public ROF dataset, it achieved strong performance on both the sunglasses and masked-face subsets, including 98.66% closed-set accuracy and 96.35% threshold-based accuracy for sunglasses and 97.92% closed-set accuracy and 95.14% threshold-based accuracy for masked faces. The low EER and high AUC values further indicate that the ArcFace-based embeddings provided strong separation between genuine and impostor scores under partial occlusion.

The real-time experiments also demonstrated that the proposed system can be used in practical scenarios. The ArcFace-based pipeline achieved the highest FPS and lowest latency on the GPU-enabled laptop and remained deployable on the Raspberry Pi 4, although with reduced speed. These findings suggest that the proposed approach is suitable for practical applications where multiple partially occluded faces may appear, such as access control, attendance monitoring, surveillance, and edge-oriented IoT environments.

The study also showed that threshold-based evaluation is important for practical recognition systems. Compared with closed-set evaluation, the 0.60 confidence threshold provides a stricter setting because uncertain predictions can be rejected as unknown instead of being forced into an incorrect identity class. The comparison with three public-code methods further showed that methods designed for specific recognition settings may not transfer equally well to broader occlusion types, different datasets, or fixed threshold-based protocols.

Future work will focus on expanding the dataset to include greater age variation, different camera types, and wider viewing angles. In addition, the system will be further optimized to improve performance on Raspberry Pi and other edge devices.

## Figures and Tables

**Table 1:** Publications on multi-face recognition and partially occluded face recognition with related attributes.

Publications	Multi-Face Recognition	Partially Occluded Face Recognition	Reported Accuracy ≥ 95%	Hybrid Pipeline	Same Dataset & Protocol	Linear SVM for Classification
[10]	✓	✗	✓	✗	✗	✗
[11]	✗	✓	✓	✓	✗	✗
[20]	✗	✓	✓	✓	✗	✗
[16]	✗	✓	✓	✓	✗	✗
[17]	✗	✓	✓	✗	✗	✗
[26]	✗	✓	✓	✗	✗	✗
[27]	✗	✓	✓	✓	✗	✗
[28]	✗	✓	✓	✗	✗	✗
[29]	✗	✓	N/A	✗	✗	✗
[30]	✗	✓	✓	✓	✗	✗
[31]	✗	✓	✓	✗	✗	✗
[32]	✗	✓	✓	✗	✗	✗
[33]	✗	✓	✓	✗	✗	✗
[34]	✗	✓	✓	✗	✗	✗
[35]	✗	✓	✓	✗	✗	✗
[36]	✗	✓	✓	✗	✗	✗
[37]	✗	✓	N/A	N/A	✗	✗
This work	✓	✓	✓	✓	✓	✓

Note: ✓ indicates that the corresponding attribute is addressed in the publication, ✗ indicates that it is not addressed, and N/A indicates that the information is not reported in the publication.

**Table 2:** Laptop specifics used in the experiments.

Component	Specification
Laptop model	ASUS X705UDR
CPU	Intel Core i7-8550U, 4 cores/8 threads
RAM	8 GB
GPU	NVIDIA GeForce GTX 1050
Operating system	Windows 11 Pro, 64-bit

**Table 3:** Raspberry Pi 4 specifications used for edge-device real-time testing.

Component	Specification
Device	Raspberry Pi 4 Model B
Processor	Quad-core CPU
Memory (RAM)	4 GB
Storage	Attached storage
Cooling	Passive cooling
Camera Interface	External USB webcam

**Table 4:** Webcam used for real-time testing in both devices.

Component	Specification
Webcam model	Redragon HITMAN GW800
Type	External USB webcam
Resolution	Full HD 1080p
Connection	USB

**Table 5:** Custom dataset distribution after preprocessing.

Subject	Training Images	Testing Images
Afrim	351	1392
Anisa	344	1379
Arjeta	352	1379
Blendi	349	1381
Dineta	335	1280
Eronit	347	1333
Erza	348	1387
Inara	331	1373
Joni	348	1359
Juliano	354	1312
Krenare	350	1404
Kristi	357	1355
Laura	338	1380
Rina	348	1404
Shkembi	318	1258
Total	5170	20,376

**Table 6:** Offline performance on the custom partially occluded face dataset.

Method	Setting	Acc.	Prec.	Rec.	F1	AUC	EER
ResNet29 + SVM	Closed-set	96.90%	96.99%	96.98%	96.98%	–	–
	Threshold	95.17%	95.29%	95.28%	95.28%	95.32%	0.0120
ConvNeXt-Tiny + SVM	Closed-set	87.63%	90.00%	79.40%	83.00%	–	–
	Threshold	85.65%	87.00%	78.40%	81.00%	68.88%	0.2332
ArcFace ResNet100 + SVM	Closed-set	99.55%	99.57%	99.56%	99.21%	–	–
	Threshold	99.20%	99.22%	99.21%	99.21%	99.33%	0.0092
Kumar et al. [9]	Closed-set	18.00%	17.95%	18.00%	17.97%	–	–
	Threshold	0.00%	0.00%	0.00%	0.00%	42.21%	0.6012
Alansari et al. [10]	Closed-set	85.45%	84.10%	85.30%	85.10%	–	–
	Threshold	65.45%	66.30%	66.28%	65.70%	88.22%	0.0230
Li et al. [11]	Closed-set	81.27%	81.38%	81.38%	81.28%	–	–
	Threshold	73.16%	73.18%	73.21%	73.19%	68.18%	0.2454

**Table 7:** Open-set unknown-identity rejection performance across all evaluated pipelines. The test set contains 1100 unknown-identity images that were not included during training.

Method	Threshold	Correctly Rejected	Falsely Accepted	Unknown Rejection Rate	False Acceptance Rate
ResNet29 + SVM	0.40	813/1100	287/1100	73.91%	26.09%
ResNet29 + SVM	0.50	899/1100	201/1100	81.73%	18.27%
ResNet29 + SVM	0.60	963/1100	137/1100	87.55%	12.45%
ResNet29 + SVM	0.70	1015/1100	85/1100	92.27%	7.73%
ResNet29 + SVM	0.80	1050/1100	50/1100	95.45%	4.55%
ConvNeXt-Tiny + SVM	0.40	841/1100	259/1100	76.45%	23.55%
ConvNeXt-Tiny + SVM	0.50	932/1100	168/1100	84.73%	15.27%
ConvNeXt-Tiny + SVM	0.60	997/1100	103/1100	90.64%	9.36%
ConvNeXt-Tiny + SVM	0.70	1047/1100	53/1100	95.18%	4.82%
ConvNeXt-Tiny + SVM	0.80	1076/1100	24/1100	97.82%	2.18%
ArcFace ResNet100 + SVM	0.40	945/1100	155/1100	85.91%	14.09%
ArcFace ResNet100 + SVM	0.50	988/1100	112/1100	89.82%	10.18%
ArcFace ResNet100 + SVM	0.60	1052/1100	48/1100	95.64%	4.36%
ArcFace ResNet100 + SVM	0.70	1090/1100	10/1100	99.09%	0.91%
ArcFace ResNet100 + SVM	0.80	1100/1100	0/1100	100.00%	0.00%
Kumar et al. [9]	0.40	1100/1100	0/1100	100.00%	0.00%
Kumar et al. [9]	0.50	1100/1100	0/1100	100.00%	0.00%
Kumar et al. [9]	0.60	1100/1100	0/1100	100.00%	0.00%
Kumar et al. [9]	0.70	1100/1100	0/1100	100.00%	0.00%
Kumar et al. [9]	0.80	1100/1100	0/1100	100.00%	0.00%
Alansari et al. [10]	0.40	1100/1100	0/1100	100.00%	0.00%
Alansari et al. [10]	0.50	1100/1100	0/1100	100.00%	0.00%
Alansari et al. [10]	0.60	1100/1100	0/1100	100.00%	0.00%
Alansari et al. [10]	0.70	1100/1100	0/1100	100.00%	0.00%
Alansari et al. [10]	0.80	1100/1100	0/1100	100.00%	0.00%
Li et al. [11]	0.40	1068/1100	32/1100	97.09%	2.91%
Li et al. [11]	0.50	1090/1100	10/1100	99.09%	0.91%
Li et al. [11]	0.60	1099/1100	1/1100	99.91%	0.09%
Li et al. [11]	0.70	1099/1100	1/1100	99.91%	0.09%
Li et al. [11]	0.80	1100/1100	0/1100	100.00%	0.00%

**Table 8:** Real-time performance on the custom dataset using live-camera input.

Method	Device	Frames	Average FPS	Average Latency
SCRFD + ResNet29 + Linear SVM	GPU laptop	2560	3.70	270.15 ms/frame
	Raspberry Pi 4	1632	2.72	367.62 ms/frame
SCRFD + ConvNeXt-Tiny + Linear SVM	GPU laptop	3525	12.07	82.82 ms/frame
	Raspberry Pi 4	2256	3.76	266.24 ms/frame
SCRFD + ArcFace ResNet100 + Linear SVM	GPU laptop	16,195	29.08	34.39 ms/frame
	Raspberry Pi 4	2760	4.60	273.40 ms/frame
Alansari et al. [10]	GPU laptop	3749	4.67	214.28 ms/frame
	Raspberry Pi 4	390	0.65	1530.00 ms/frame
Li et al. [11]	GPU laptop	7966	12.66	70.21 ms/frame
	Raspberry Pi 4	2046	3.41	293.39 ms/frame
Kumar et al. [9]	–	–	Not real-time	–

**Table 9:** Runtime resource profile on the Raspberry Pi 4 Model B during real-time edge-device inference.

Method/Pipeline	CPU Usage (%)	RAM Usage (GB)	Estimated Power Usage (W)	Edge Runtime Demand
SCRFD + ResNet29 + Linear SVM	85–92	1.0–1.3	5.3–6.2	High
SCRFD + ConvNeXt-Tiny + Linear SVM	78–88	1.3–1.7	5.1–6.0	Medium–High
SCRFD + ArcFace ResNet100 + Linear SVM	72–84	1.0–1.4	4.8–5.8	Medium–High, optimized
Alansari et al. [10]	90–98	1.2–1.6	5.8–6.8	Very High
Li et al. [11]	80–90	0.9–1.3	5.0–6.0	High
Kumar et al. [9]	>95	>1.4	>6.0	Not suitable for real-time deployment

**Table 10:** Representative real-time confidence scores on the custom dataset with method components.

Method	Detector	Feature Extractor	Classifier	Single-Face	Multi-Face
Kumar et al. [9]	OpenCV DNN SSD	Modified MobileNetV2	Softmax	Not real-time	Not real-time
Alansari et al. [10]	YOLOv5Face	EfficientFaceV2S	Cosine/NN	83.876%	66.445%
Li et al. [11]	RetinaFace	ArcFace-ResNet	Cosine similarity	79.4%	71.5%
ResNet29 + SVM	SCRFD	ResNet29	Linear SVM	97.467%	95.437%
ArcFace ResNet100 + SVM	SCRFD	ArcFace ResNet100	Linear SVM	99.46%	97.475%
ConvNeXt-Tiny + SVM	SCRFD	ConvNeXt-Tiny	Linear SVM	90.4%	90.0% with 3 faces

**Table 11:** Closed-set offline performance on the ROF sunglasses subset.

Method	Accuracy	Weighted Precision	Weighted Recall	Weighted F1-Score
SCRFD + ResNet29 + Linear SVM	93.05%	94.59%	93.05%	93.21%
SCRFD + ConvNeXt-Tiny + Linear SVM	66.67%	79.95%	66.67%	73.12%
SCRFD + ArcFace ResNet100 + Linear SVM	98.66%	98.82%	98.66%	98.63%
Kumar et al. [9]	10.22%	14.35%	10.22%	7.31%
Alansari et al. [10]	98.88%	98.93%	98.88%	98.86%
Li et al. [11]	59.25%	71.83%	59.25%	60.31%

**Table 12:** Threshold-based offline performance on the ROF sunglasses subset.

Method	Tau Accuracy	Tau W-P	Tau W-R	Tau W-F1-Score	Reject	AUC	EER
SCRFD + ResNet29 + Linear SVM	90.32%	96.41%	90.32%	92.63%	5.46%	96.15%	0.0844
SCRFD + ConvNeXt-Tiny + Linear SVM	54.26%	53.41%	44.26%	46.08%	45.74%	52.00%	0.4355
SCRFD + ArcFace ResNet100 + Linear SVM	96.35%	100.00%	96.35%	98.02%	3.65%	99.44%	0.0061
Kumar et al. [9]	0.00%	0.00%	0.00%	0.00%	100.00%	66.44%	0.3914
Alansari et al. [10]	61.39%	100.00%	61.39%	72.48%	38.61%	99.25%	0.0205
Li et al. [11]	4.87%	35.64%	4.87%	7.47%	95.13%	88.13%	0.1989

**Table 13:** Closed-set offline performance on the ROF masked-face subset.

Method	Accuracy	Weighted Precision	Weighted Recall	Weighted F1-Score
SCRFD + ResNet29 + Linear SVM	72.88%	74.86%	72.88%	70.55%
SCRFD + ConvNeXt-Tiny + Linear SVM	38.06%	37.61%	38.06%	34.21%
SCRFD + ArcFace ResNet100 + Linear SVM	97.92%	98.17%	97.92%	97.85%
Kumar et al. [9]	22.22%	25.15%	22.22%	18.91%
Alansari et al. [10]	96.44%	96.71%	96.44%	98.40%
Li et al. [11]	51.39%	58.04%	51.39%	50.58%

**Table 14:** Threshold-based offline performance on the ROF masked-face subset.

Method	Tau Accuracy	W-Precision	W-Recall	W-F1	Reject	AUC	EER
SCRFD + ResNet29 + Linear SVM	64.24%	79.87%	64.24%	68.13%	32.37%	78.74%	0.1881
SCRFD + ConvNeXt-Tiny + Linear SVM	18.06%	17.61%	18.06%	14.21%	81.94%	42.31%	0.3191
SCRFD + ArcFace ResNet100 + Linear SVM	95.14%	100.00%	95.14%	97.31%	4.86%	99.66%	0.0057
Kumar et al. [9]	0.00%	0.00%	0.00%	0.00%	100.00%	70.97%	0.3468
Alansari et al. [10]	18.75%	54.69%	18.75%	25.90%	81.25%	98.85%	0.0269
Li et al. [11]	0.00%	0.00%	0.00%	0.00%	100.00%	84.03%	0.2425

**Table 15:** Additional threshold-sensitivity analysis across the custom dataset and ROF subsets for all evaluated pipelines. The 0.60 operating-point results are reported in the main performance tables for the ROF dataset and are therefore not repeated here; they are repeated only for the custom dataset, where the reject rate was not included in the main table.

Dataset/Subset	Method	Threshold	Accuracy	W-F1	Reject Rate	EER
Custom	ResNet29 + SVM	0.40	96.50%	96.55%	1%	0.0120
Custom	ResNet29 + SVM	0.50	96.10%	96.20%	1.81%	0.0120
Custom	ResNet29 + SVM	0.60	95.17%	95.28%	3.12%	0.0120
Custom	ResNet29 + SVM	0.70	93.6%	93.8	5.2%	0.0120
Custom	ResNet29 + SVM	0.80	90.54%	90.49%	8.57%	0.0120
Custom	ConvNeXt-Tiny + SVM	0.40	87.22%	82.74%	1.54%	0.2332
Custom	ConvNeXt-Tiny + SVM	0.50	85.65%	81.23%	2.82%	0.2332
Custom	ConvNeXt-Tiny + SVM	0.60	85.65%	80.12%	9.12%	0.0120
Custom	ConvNeXt-Tiny + SVM	0.70	82.50%	78.20%	18.51%	0.2332
Custom	ConvNeXt-Tiny + SVM	0.80	77.23%	72.82%	21.23%	0.2332
Custom	ArcFace ResNet100 + SVM	0.40	99.5%	99.51%	0.15%	0.0092
Custom	ArcFace ResNet100 + SVM	0.50	99.36%	99.42%	0.48%	0.0092
Custom	ArcFace ResNet100 + SVM	0.60	99.2%	99.21%	0.8%	0.0092
Custom	ArcFace ResNet100 + SVM	0.70	98.75%	98.78%	1.25%	0.0092
Custom	ArcFace ResNet100 + SVM	0.80	98.2%	98.25%	1.8%	0.0092
Custom	Kumar et al. [9]	0.40	7.2%	5.8%	74%	0.6012
Custom	Kumar et al. [9]	0.50	2.4%	1.8%	91%	0.6012
Custom	Kumar et al. [9]	0.60	0%	0%	100%	0.6012
Custom	Kumar et al. [9]	0.70	0%	0%	100%	0.6012
Custom	Kumar et al. [9]	0.80	0%	0%	100%	0.6012
Custom	Alansari et al. [10]	0.40	82.5%	82.8%	12%	0.0230
Custom	Alansari et al. [10]	0.50	76.5%	76.7%	22%	0.0230
Custom	Alansari et al. [10]	0.60	65.45%	65.7%	34.55%	0.0230
Custom	Alansari et al. [10]	0.70	50.32%	50.87	49.68%	0.0230
Custom	Alansari et al. [10]	0.80	44.72%	45.65%	55.28%	0.0230
Custom	Li et al. [11]	0.40	79.22%	79.11%	7.27%	0.2454
Custom	Li et al. [11]	0.50	76.63%	76.37%	14.89%	0.2454
Custom	Li et al. [11]	0.60	73.16%	73.19%	26.84%	0.2454
Custom	Li et al. [11]	0.70	65.42%	65.68%	34.58%	0.2454
Custom	Li et al. [11]	0.80	55.92%	56.13%	44.08%	0.2454
ROF Sunglasses	ResNet29 + SVM	0.40	93.05%	93.21%	4.79%	0.0844
ROF Sunglasses	ResNet29 + SVM	0.50	92.31%	93.01%	5.49%	0.0844
ROF Sunglasses	ResNet29 + SVM	0.70	85.86%	90.04%	14.14%	0.0844
ROF Sunglasses	ResNet29 + SVM	0.80	79.9%	85.99%	20.1%	0.0844
ROF Sunglasses	ConvNeXt-Tiny + SVM	0.40	60.02%	50.83%	28.22%	0.4355
ROF Sunglasses	ConvNeXt-Tiny + SVM	0.50	57.99%	54.32%	38.45%	0.4355
ROF Sunglasses	ConvNeXt-Tiny + SVM	0.70	44.65%	41.54%	55.35%	0.4355
ROF Sunglasses	ConvNeXt-Tiny + SVM	0.80	28.67%	23.67%	71.33%	0.4355
ROF Sunglasses	ArcFace ResNet100 + SVM	0.40	98.05%	98.94%	1.95%	0.0061
ROF Sunglasses	ArcFace ResNet100 + SVM	0.50	97.93%	98.87%	2.07%	0.0061
ROF Sunglasses	ArcFace ResNet100 + SVM	0.70	94.40%	96.89%	5.60%	0.0061
ROF Sunglasses	ArcFace ResNet100 + SVM	0.80	90.79%	96.84%	9.21%	0.0061
ROF Sunglasses	Kumar et al. [9]	0.40	7.18%	5.77%	74.12%	0.3914
ROF Sunglasses	Kumar et al. [9]	0.50	6.45%	5.35%	88.47%	0.3914
ROF Sunglasses	Kumar et al. [9]	0.70	0%	0%	100%	0.3914
ROF Sunglasses	Kumar et al. [9]	0.80	0%	0%	100%	0.3914
ROF Sunglasses	Alansari et al. [10]	0.40	96.76%	98.26%	3.24%	0.0205
ROF Sunglasses	Alansari et al. [10]	0.50	88.17%	93.28%	11.83%	0.0205
ROF Sunglasses	Alansari et al. [10]	0.70	20.05%	28.6%	79.95%	0.0205
ROF Sunglasses	Alansari et al. [10]	0.80	4.36%	6.77%	95.64%	0.0205
ROF Sunglasses	Li et al. [11]	0.40	42.94%	54.32%	51.7%	0.1989
ROF Sunglasses	Li et al. [11]	0.50	16.79%	25.23%	83.09%	0.1989
ROF Sunglasses	Li et al. [11]	0.70	1.09%	1.9%	98.91%	0.1989
ROF Sunglasses	Li et al. [11]	0.80	0.36%	0.66%	99.64%	0.1989
ROF Mask	ResNet29 + SVM	0.40	71.88%	69.55%	17.14%	0.1881
ROF Mask	ResNet29 + SVM	0.50	70.69%	68.91%	21.45%	0.1881
ROF Mask	ResNet29 + SVM	0.70	62.10%	66.73%	35.03%	0.1881
ROF Mask	ResNet29 + SVM	0.80	60.41%	64.90%	37.42%	0.1881
ROF Mask	ConvNeXt-Tiny + SVM	0.40	23.18%	22.32%	52.32%	0.3191
ROF Mask	ConvNeXt-Tiny + SVM	0.50	21.98%	20.54%	73.28%	0.3191
ROF Mask	ConvNeXt-Tiny + SVM	0.70	4.3%	3.27%	95.7%	0.3191
ROF Mask	ConvNeXt-Tiny + SVM	0.80	1.2%	0.92%	99.2%	0.3191
ROF Mask	ArcFace ResNet100 + SVM	0.40	97.22%	98.42%	2.78%	0.0057
ROF Mask	ArcFace ResNet100 + SVM	0.50	95.83%	97.67%	4.17%	0.0057
ROF Mask	ArcFace ResNet100 + SVM	0.70	87.08%	98.08%	12.92%	0.0057
ROF Mask	ArcFace ResNet100 + SVM	0.80	82.08%	83.31%	17.92%	0.0057
ROF Mask	Kumar et al. [9]	0.40	14.58%	14.41%	40.28%	0.3468
ROF Mask	Kumar et al. [9]	0.50	10.42%	12.67%	61.11%	0.3468
ROF Mask	Kumar et al. [9]	0.70	0%	0%	100%	0.3468
ROF Mask	Kumar et al. [9]	0.80	0%	0%	100%	0.3468
ROF Mask	Alansari et al. [10]	0.40	90.62%	94.45%	8.59%	0.0269
ROF Mask	Alansari et al. [10]	0.50	64.84%	73.57%	35.16%	0.0269
ROF Mask	Alansari et al. [10]	0.70	0%	0%	100%	0.0269
ROF Mask	Alansari et al. [10]	0.80	0%	0%	100%	0.0269
ROF Mask	Li et al. [11]	0.40	15.28%	22.77%	84.03%	0.2425
ROF Mask	Li et al. [11]	0.50	2.08%	3.7%	97.92%	0.2425
ROF Mask	Li et al. [11]	0.70	0%	0%	100%	0.2425
ROF Mask	Li et al. [11]	0.80	0%	0%	100%	0.2425

## Data Availability

The ROF dataset is publicly available at https://github.com/ekremerakin/RealWorldOccludedFaces (accessed on 16 March 2026). The custom partially occluded face dataset is not publicly available due to participant privacy and consent restrictions, as it contains facial images used for biometric recognition experiments. During dataset preparation, personal identifiers were removed where possible, and participants were stored using internal labels or participant codes. In the real-time system, first-name labels were used only for internal experimental visualization and were not intended as public identifiers. Any examples included in the manuscript are shown in blurred form, and identifiable information is minimized to protect participant privacy. Access to the custom dataset may be granted by the corresponding author upon reasonable request, strictly for academic research purposes, subject to appropriate ethical approval, data-use conditions, and consistency with the consent provided by the participants or their legal guardians.
